# Prevalence of *c-KIT* Mutations in Gonadoblastoma and Dysgerminomas of Patients with Disorders of Sex Development (DSD) and Ovarian Dysgerminomas

**DOI:** 10.1371/journal.pone.0043952

**Published:** 2012-08-28

**Authors:** Remko Hersmus, Hans Stoop, Gert Jan van de Geijn, Ronak Eini, Katharina Biermann, J. Wolter Oosterhuis, Catharina DHooge, Dominik T. Schneider, Isabelle C. Meijssen, Winand N. M. Dinjens, Hendrikus Jan Dubbink, Stenvert L. S. Drop, Leendert H. J. Looijenga

**Affiliations:** 1 Department of Pathology, Erasmus MC - University Medical Center Rotterdam, Josephine Nefkens Institute, Daniel den Hoed Cancer Center, Rotterdam, The Netherlands; 2 Department of Pediatrics, Division of Pediatric Hemato-Oncology, University Hospital Ghent, Ghent, Belgium; 3 Clinic of Pediatrics, Municipal Hospital Dortmund, Dortmund, Germany; 4 Molecular Diagnostics, Department of Pathology, Erasmus MC - University Medical Center Rotterdam, Josephine Nefkens Institute, Rotterdam, The Netherlands; 5 Department of Pediatric Endocrinology, Erasmus MC - University Medical Center Rotterdam, Sophia Children’s Hospital, Rotterdam, The Netherlands; Florida International University, United States of America

## Abstract

Activating *c-KIT* mutations (exons 11 and 17) are found in 10–40% of testicular seminomas, the majority being missense point mutations (codon 816). Malignant ovarian dysgerminomas represent ∼3% of all ovarian cancers in Western countries, resembling testicular seminomas, regarding chromosomal aberrations and *c-KIT* mutations. DSD patients with specific Y-sequences have an increased risk for Type II Germ Cell Tumor/Cancer, with gonadoblastoma as precursor progressing to dysgerminoma. Here we present analysis of *c-KIT* exon 8, 9, 11, 13 and 17, and *PDGFRA* exon 12, 14 and 18 by conventional sequencing together with mutational analysis of *c-KIT* codon 816 by a sensitive and specific LightCycler melting curve analysis, confirmed by sequencing. The results are combined with data on TSPY and OCT3/4 expression in a series of 16 DSD patients presenting with gonadoblastoma and dysgerminoma and 15 patients presenting pure ovarian dysgerminomas without DSD. *c-KIT* codon 816 mutations were detected in five out of the total of 31 cases (all found in pure ovarian dysgerminomas). A synonymous SNP (rs 5578615) was detected in two patients, one DSD patient (with bilateral disease) and one patient with dysgerminoma. Next to these, three codon N822K mutations were detected in the group of 15 pure ovarian dysgerminomas. In total activating *c-KIT* mutations were found in 53% of ovarian dysgerminomas without DSD. In the group of 16 DSD cases a N505I and D820E mutation was found in a single tumor of a patient with gonadoblastoma and dysgerminoma. No *PDGFRA* mutations were found. Positive OCT3/4 staining was present in all gonadoblastomas and dysgerminomas investigated, TSPY expression was only seen in the gonadoblastoma/dysgerminoma lesions of the 16 DSD patients. This data supports the existence of two distinct but parallel pathways in the development of dysgerminoma, in which mutational status of *c-KIT* might parallel the presence of TSPY.

## Introduction

c-KIT belongs to the Type III tyrosine kinase receptor family, which also includes the platelet-derived growth factor receptor (PDGFR) and macrophage-colony stimulating receptor (M-CSFR). The ligand for c-KIT is the stem cell factor (SCF, KITLG) and the SCF-KIT pathway regulates the differentiation of melanocytes, red blood cells, mast cells, interstitial cells of Cajal, and germ cells [1], [2], [3]. Moreover, this pathway is important in the survival of primordial germ cells (PGCs) [4], [5]. Expression of c-KIT and gain-of-function mutations in *c-KIT* has been found in mastocytosis, leukemia and gastro-intestinal stromal tumors (GIST) [6], [7], [8]. In GIST activating mutations in *c-KIT* exons 8, 9, 11, 13 and 17 are found in 75–80% of cases, mutations in *PDGFRA* exons 12, 14 and 18 in 5–8%, and they are mutually exclusive (for review [9]).

Activating *c-KIT* mutations have also been found in human germ cell tumors/cancers (GCC), and 10–40% of testicular seminomas harbor activating mutations in exons 11 and 17. About two thirds are missense point mutations at codon 816 [2], [10], [11], [12], which are also found in almost all mast cell tumors [13]. Noteworthy is that activating *c-KIT* mutations have been found in a subset of tumors showing the same histology as testicular seminoma, namely; mediastinal seminomas, intracranial germinomas and ovarian dysgerminomas [14], [15], [16]. Next to mutations in c-KIT, amplification of chromosome 4q12, harboring the *c-KIT* gene, has been described in testicular GCC, likely related to the progression to seminoma [10]. Malignant ovarian dysgerminomas represent approximately 3% of all ovarian cancers in Western countries, and share a morphological resemblance, and show a similar pattern of chromosomal aberrations [17] with testicular GCC. Families with both ovarian and testicular GCC have been reported, suggestive of a common etiology [18].

Disorders of Sex Development (DSD), previously referred to as intersex, are a congenital condition in which there is an atypical development of the chromosomal, gonadal or anatomical sex [19]. DSD patients with gonadal dysgenesis or hypovirilization harboring Y-chromosomal material in their karyotype have an increased risk of developing GCC (for review [20], [21]). The precursor lesion in the dysgenetic gonads of these patients is the gonadoblastoma (GB), or carcinoma *in situ* (CIS), depending on the level of gonadal testicularization [22]. The invasive component is the dysgerminoma in most cases (genetically the counterpart of the seminoma of the testis). For the development of GB the presence of the GonadoBlastoma locus on the Y-chromosome (GBY) is imperative, with the testis specific protein, Y-linked (*TSPY)* gene being the most likely candidate in this region. TSPY expression is linked to the proliferation and survival of germ cells, and expression is increased in CIS, GB and sometimes seminoma [23]. The octamer-binding protein 3 (OCT3/4, POU5F1) is specifically expressed in all GCC with pluripotent potential, as well as in the neoplastic precursor lesions CIS and GB [24], [25]. Germ cells residing in an unfavorable environment, as is the case in DSD, might escape cell death by prolonged expression of both OCT3/4 and TSPY. If mutations in *c-KIT* or *PDGFRA* play a significant role in the development of GB and the development of dysgerminoma in DSD patients is not clear so far because of the lack of multiple studies.

Here we report the analysis of activating mutations in codon 816 of *c-KIT* in 31 patients with a GB and/or dysgerminoma by LightCycler analysis, together with conventional sequence analysis of *c-KIT* exons 8, 9, 11, 13 and 17, and *PDGFRA* exons 12, 14 and 18, mutations in which are frequently found in GIST. These results are linked with karyotype, histology of the gonads, expression of TSPY in the tumors and putative role of the mutations found in the etiology of the disease.

## Materials and Methods

### Tissue Samples and Immunohistochemistry

In total 31 cases, consisting of eleven cases of GB, fifteen cases of DG and eight cases of GB with DG were retrieved from the archives (Table 1). Collected tissue samples were diagnosed according to WHO standards [26] by an experienced pathologist (JWO). Use of tissue samples for scientific reasons was approved by the Medical Ethical Committee ErasmusMC (MEC 02.981 and CCR2041). Patients gave their verbal consent that left over material, after a diagnostic procedure, can be used for scientific purposes. This agreement is not documented, as agreed upon by the MEC. If patients chose to not consent, it is specifically indicated in the clinical files, and samples were excluded. This consent procedure was used according to the “Code for Proper Secondary Use of Human Tissue in the Netherlands.”

**Table 1:** Overview mutations found and immunohistochemistry.

					c-KIT LightCycler		c-KIT sequencing						PDGFRA sequencing							Immunohistochemistry gonad					
Case No	Age	Sex	Malignancy	Karyotype	c-KIT LC	Seq. LC Products	Ex 8	Ex 9a	Ex 9b	Ex 11	Ex 13	Ex 17	Ex 12a	Ex 12b		Ex 14		Ex 18		c-KIT (CD117)		OCT3/4		TSPY	
**1**	16	F	GB	46XY	shift		ND	ND	ND	ND	ND	ND	ND	ND		ND		ND		**+**		**+**		**+**	
**1** §	16	F	GB	46XY	shift				ND				P567P	P567P						**+**		**+**		**+**	
**2** §	19	F	GB	46XY	shift				ND				ND	ND						**+**		**+**		**+**	
**2** §	19	F	GB/DG	46XY	shift		ND	ND	ND	ND	ND	ND	ND	ND		ND		ND		**+**		**+**		**+**	
**3** §	22	F	GB	46XY		ND	ND	ND	ND	ND	ND	ND	ND	ND		ND		ND		**+**		**+**		**+**	
**3**	22	F	GB/DG/YST/imTE	46XY		ND			ND	ND			ND	ND		ND		ND		**+**		**+**		**+**	
**4**	21	M	GB/DG/CIS	46XY	shift	I798I			ND				ND							**+/−**		**+**		**+**	
**5** §	9	F	GB/TE/YST	46XY		ND			ND			ND	P567P	P567P						**+ (few cells)**		ND		**+**	
6§	14	F	GB	46XY		ND							P567P	P567P						**−**		**+ (few cells)**		**+ (few cells)**	
7§	2	F	GB	46XY		ND							P567P	P567P		ND		ND		**+**		**−**		**−**	
8	26	M	GB/CIS/itSE	46XY		ND							P567P	P567P						**+**		**+**		**+**	
9	14	F	GB/DG	46XY		ND							P567P	P567P						**+**		**+**		**+**	
10	18	F	GB/DG	46XY		ND			ND				P567P	P567P						**+**		**+**		**+**	
11	22	F	GB/DG	46XY		ND							P567P	P567P						**+**		**+**		**+**	
12§	1	M	GB	45X/46XY		ND						ND	ND	ND	ND				**+**		**+**		**+**		
13§	17	F	GB	45X/46XY		ND							P567P	P567P						**−**		**+ (few cells)**		**−**	
14	20	F	GB	45X/46XY		ND			ND				P567P	P567P						**+**		**+**		**+**	
15§	3m	F	GB	46XX		ND							P567P	P567P						**+**		**+**		**+ (few cells)**	
16	36	M	GB/DG	46XY‡		ND			**N505I**			**D820E**	P567P	P567P						**+**		**+**		**+**	
17	14	NA	DG	NA	**His**	**D816H**						**D816H**	P567P	P567P						**+**		**+**		**−**	
18	19	NA	DG	NA		ND							P567P	P567P						**+**		**+**		**−**	
19	17	NA	DG	NA	**Val**	**D816V**						**D816V**	P567P	P567P						**+**		**+**		**−**	
20	NA	NA	DG	NA		ND							P567P	P567P						**+**		**+**		**−**	
21	19	NA	DG	NA		ND						**N822K**	P567P	P567P						**+**		**+**		**−**	
22	15	F	DG	NA	**Val**	**D816V**						**D816V**	P567P	P567P						**+**		**+**		**−**	
23	15	F	DG	46XX		ND			ND	ND		ND	ND	ND		ND		ND		ND		**+**		ND	
24	12	F	DG	46XX		ND						**N822K**	P567P	P567P						**+**		**+**		**−**	
25	7	F	DG	46XX	**Tyr**	**D816Y**						**D816Y**	P567P	P567P						ND		**+**		**−**	
26	16	F	DG	46XX		ND							P567P	P567P						**+/−**		**+**		**−**	
27	14	F	DG	46XX		ND			ND			**N822K**	P567P	P567P						**+**		**+**		**−**	
28	14	F	DG	46XX		ND						I798I	P567P	P567P						**+/−**		**+**		**−**	
29	16	NA	DG	46XX		ND							P567P	P567P						ND		**+**		**−**	
30	6	F	DG/TE	46XX		ND							P567P	P567P						**+**		**+**		**−**	
31	10	F	DG/YST	46XX		ND						**D816V**	P567P	P567P						ND		**+**		**−**	

Ex, exon; ND, not done; NA, not available; mutations indicated in bold, other samples wild type unless indicated otherwise; DG, dysgerminoma; GB, gonadoblastoma; CIS, carcinoma in situ; TE, teratoma (im: immature); YST yolk sac tumor; itSE, intratubular seminoma;

seq, sequence; LC, LightCycler; c-KIT LC, LightCycler melting curve results; case numbers in bold are bilateral cases.

§ tumor/precursor percentage below 50%.

‡ as determined by FISH on gonadal tissue.

I789I: heterozygous synonymous SNP, rs.5578615.

P567P: homozygous synonymous SNP, rs.1873778.

Immunohistochemistry was performed on paraffin-embedded tissue sections of 3-µm thickness. After deparaffinization and 5 min. incubation in 3% H_2_O_2_ to inactivate endogenous peroxidase activity, antigen retrieval was carried out by heating under pressure of up to 1.2 bar in an appropriate buffer; 0.01 M sodium citrate (pH 6) or 0.01 M EGTA, 0.01 M TRIS (pH 9). After blocking endogenous biotin using the avidin/biotin blocking kit (SP-2001, Vector Laboratories, Burlingame, CA, USA), the sections were incubated for either 2 hrs at room-temperature (OCT3/4, c-KIT (CD117) or overnight at 4°C (TSPY). Appropriate biotinylated secondary antibodies were used for detection and were visualized using the avidin-biotin detection and substrate kits (Vector Laboratories). The antibodies used directed against OCT3/4, TSPY and c-KIT have been described before [27], [28], [29].

### DNA Isolation and c-KIT Codon 816 Mutational Screen

DNA was isolated from formalin-fixed-paraffin-embedded material using a standard protocol, percentage of tumor present in each sample was over 50% unless indicated otherwise (Table 1). In brief, 10 slices of 10-µm thickness were cut and incubated three times with xylene for at least 30 min at RT, after which the pellet was washed each time with ethanol. Lysisbuffer consisting of 10 mM TRIS, 100 mM NaCl, 5 mM EDTA, 1% SDS and 1 mM CaCl_2_ together with 10 mg/ml proteinase-K was added, and the sample was incubated for 16 hrs at 50°C, while shaking at 1200 rpm. DNA was subsequently extracted by standard phenol/chloroform extraction and ethanol precipitation. DNA was dissolved in 10 mM TRIS with 1 mM EDTA. DNA quality and concentration was checked on the Nanodrop 1000 (ThermoScientific, Wilmington, DE, USA).

50 ng of DNA from each sample was screened for c-KIT D816V, D816H, D816Y mutations using a melting-curve based LightCycler assay (Roche Diagnostics, Mannheim, Germany) with forward primer KIT816For, CAGCCAGAAATATCCTCCTTACT; or KIT816 ForA, CTTTTCTCCTCCAACCTAATAG; reverse primer KIT816Rev, TTGCAGGACTGTCAAGCAGAG; and hybridization probes c-KIT-anchor, LC640-ATGTGGTTAAAGGAAACGTGAGTACCCA–PH; c-KIT-sensor VAL, AGCCAGAGTCATCAAGAATGATTCTA–FL; c-KIT-sensor TYR, AGCCAGACACATCAAGAATGATTCTA–FL; c-KIT-sensor HIS, AGCCAGATACATCAAGAATGATTCTA. To suppress wild type sequences, all reactions were performed with and without addition of a locked nucleic acid (LNA), c-KIT probe GCCAGAGACATCAAGAATG (all primers produced by TIB molbiol, Berlin, Germany). Mixing experiments showed that with the addition of LNA to block wild type sequence, the lower limit of detection was 20 fg of mutant DNA in 50 ng of wild type DNA, and routinely 20 pg of mutant DNA could be detected (data not shown). As a control, samples containing the c.816 mutation under investigation were included in each experiment and were analyzed with and without LNA, together with the experimental samples. The PCR reaction was carried out in a 20 µL volume with 0.5 µM each of forward, reverse, anchor and appropriate sensor probe, 0.01 µM of LNA, 3 mM MgCl_2_ and 2 µL LightCycler Fast-Start DNA Master HybProbe mix. Reactions were run on a LightCycler Instrument (Roche Diagnostics, Almere, The Netherlands). Amplification was performed with 45 cycles using 60°C annealing temperature. Final melting curve analysis was started at 40°C up to 95°C with a slope of 0.2°C /second and continuous detection with channel F2/F1. Lightcycler data was analyzed using the LightCycler 3.0 software (Roche Diagnostics). Samples showing an aberrant melting curve were run at least in duplicate.

### Sequence Analysis

All cases found to be positive in the c-KIT c.816 screen were confirmed by sequence analysis. Approximately 100 ng of PCR product was treated with ExoSAP-IT (GE Healthcare Life Sciences, Piscataway, NJ, USA) following manufacturers instructions, and directly sequenced with 3.3 pmol of each forward and reverse primer using the Big Dye terminator Cycle Sequencing Kit (Applera, Darmstadt, Germany). After initial denaturation at 95°C for 5 min, 25 cycles at 94°C for 15 seconds and 60°C for 4 minutes were performed. Sequence analysis was performed on an ABI 3100 Genetic Analyzer (Applied Biosystems, Foster City, CA, USA).

In addition to screening for activating mutations of c.816, in all samples *c-KIT* exon 8, 9, 11, 13, en 17 and *PDGFRA* exon 12, 14 and 18 were analyzed by conventional bidirectional cycle sequencing of PCR-amplified fragments. Amplification of 50 ng genomic DNA of each sample was performed with M13-tailed primers (Table S1). After initial denaturation at 95°C for 3 min, 35 cycles of 95°C for 30 seconds, 60°C for 45 seconds, and 72°C for 45 seconds were performed, followed by 10 min at 72°C. Subsequent sequence analyses of the PCR products was carried out with M13 forward and reverse primers, essentially as described above.

## Results

The mean age of diagnosis of the GB and/or DG was 15 years (range, 3 months-36 years, see Table 1). The mean age of diagnosis between the group of patients with DSD (cases 1–16), being 16 (3 months-36 years) and the group with ovarian dysgerminoma (cases 17–31), being 14 (6–19 years) did not differ significantly. Within the group of DSD patients the mean age of diagnosis did differ between patients showing GB, being 13 and patients who had a dysgerminoma with GB, being 21 years. In total, twenty-two cases showed a dysgerminoma component; thirteen patients had pure dysgerminoma, three patients had non-dysgerminoma components (yolk sac tumor and (immature) teratoma) next to the dysgerminoma component, and six patients showed GB next to dysgerminoma. One patient showed teratoma and yolk sac tumor next to GB. Eight patients did not have an invasive component; seven showed GB (one bilaterally) and one patient had GB next to CIS and intratubular seminoma. Five cases presented with bilateral disease; one case showing GB in both gonads, one patient having GB in one gonad and GB together with dysgerminoma in the other, one case with GB in one gonad and GB next to dysgerminoma, yolk sac tumor and immature teratoma in the other, and from two cases only material from one of the gonads was available, showing GB, CIS, and dysgerminoma in one patient and GB, teratoma and yolk sac tumor in the other patient (cases 1–5, Table 1).

LightCycler analysis detected variants in exon 17 of *c-KIT* in five out of the total group of 31 patients (19%). Four were found in the group of ovarian dysgerminomas (27%: four out of fifteen cases), consisting of two D816V, one D816H and one D816Y mutation (cases 17, 19, 22 and 25, Table 1). All mutations at codon 816 were detected in the LightCycler assay, in analyses with and without LNA added, showing a shift in melting curves which were compared with control samples (Figure S1). One variant in *c-KIT* exon 17 was found in the group of DSD patients, which changed the codon 178 sequence from ATC to ATT (I798I), which encodes a known synonymous SNP (rs. 55789615) (case 4, Table 1). This variation shifted the melting curve to a position different from that of any of the control mutation samples included (data not shown). Two other samples produced an aberrant melting curve (case 1 and 2, Table 1), but no mutation was detected in subsequent sequencing, despite analyzing the samples in triplicate for all three c.816 variants on two independent DNA isolations (data not shown). All mutations found were verified by sequencing the LightCycler products from reactions with and without LNA (Figure S1). All other samples tested showed melting curves identical to non-mutated Asp 816 (data not shown).

Next to the c.816 LightCycler analysis, conventional Sanger sequencing of *c-KIT* exons 8, 9, 11, 13, and 17 was performed on the DNA samples in a diagnostic setting. This confirmed the presence of the four c.816 mutations found by the LightCycler analysis (cases 17, 19, 22 and 25, Table 1), but also revealed an additional D816V mutation (case 31, Table 1). Furthermore, in three ovarian dysgerminoma cases a N822K mutation was found (cases 21, 24 and 27 Table 1). In total, in eight out of fifteen ovarian dysgerminoma cases (53%) an exon 17 mutation was found. One patient (case 28, Table 1), showed a heterozygous synonymous SNP (rs. 55789615). In case 16 a D820E mutation in exon 17, next to a N505I mutation in exon 9 was found, being the only DSD case showing mutations in *c-KIT* (6%, 1 out of 16). No mutations in any of the other exons analyzed were found. Sequence analysis of *PDGFRA* exon 12, 14 and 18 did not reveal any mutations, only a homozygous synonymous SNP in exon 12 (rs. 1873778) was detected in all samples analyzed.

Immunohistochemical analysis of c-KIT showed no correlation between the presence of *c-KIT* activating mutations and protein expression in the tumor. In four cases c-KIT immunohistochemistry was not investigated (case 23, 25, 29 and 31), as no additional material was available (Table 1). Staining for c-KIT was variable in the whole series analyzed, ranging from absent through intermediate to strong staining and no clear difference between the DSD and ovarian dysgerminoma subgroups could be seen. As expected, staining for OCT3/4 was positive in the GB, and dysgerminoma components in all cases analyzed, with the exception of case 7. TSPY staining correlated with the two subgroups of patients analyzed, being positive in the DSD group (cases 1–16), with the exception of cases 7 and 13, which showed no staining, and negative in the ovarian dysgerminomas (cases 17–31) (p-value 3.6×10^−8^).

## Discussion

c-KIT expression has been demonstrated in a wide variety of human tumors, although in most types expression is variable. The highest percentages are seen in gastro-intestinal tumors, seminomas, adenoid-cystic carcinomas and malignant melanomas, and amplification and enhanced expression is associated with seminoma progression [10], [30]. The presence of activating mutations of *c-KIT* in testicular seminomas is well known. Although ovarian dysgerminomas resemble seminomas in morphology and chromosomal aberrations [31], expression of c-KIT is not extensively explored. Here we analyzed fifteen cases of pure ovarian dysgerminomas and found that c-KIT is expressed, although variable, in all but two of the cases analyzed. Mutations in *c-KIT* codon 816 were found in 5 (33%) and mutations in codon 822 in 3 (20%) out of the 15 pure ovarian dysgerminoma cases (case 17, 19, 22, 25, 31 and 21, 24, 27 respectively, Table 1), accounting for 53% of cases analyzed. No mutations were detected in c-KIT exon 8, 9, 11, and 13. Although most ovarian dysgerminomas express c-KIT, we could not find a correlation between expression and *c-KIT* exon 17 mutations. It is known that in GIST in addition to mutations in *c-KIT*, also mutations in *PDGFRA* exon 12, 14 and 18 play a role and that these are mutually exclusive [9]. Sequencing *PDGFRA* did not reveal mutations in any of the dysgerminoma DNA samples analyzed, only a variation in exon 12 was found in almost all cases (homozygous synonymous SNP, rs. 1873778, Table 1). This indicates that mutations in *PDGFRA* do not play a major role in the development of (ovarian) dysgerminomas or GB. The results shown here extend those of Cheng *et al.* and Hoei-Hansen *et al.* [16], [32]. Cheng *et al.* [32] analyzed 22 cases of dysgerminoma and found a *c-KIT* codon 816 mutation in 27% of cases, and KIT expression in 87%. Hoei-Hansen *et al.* found c-KIT codon 816 mutations in five out of seventeen dysgerminoma cases (29%) with 80% expressing c-KIT [16]. Furthermore, also in gastro-intestinal tumors *KIT* mutation rate is lower than the expression rate of KIT [33], [34]. The results presented here suggest that in about half of ovarian dysgerminomas activating mutations in *c-KIT* play a role, with about a third consisting of codon 816 mutations, as has been reported by others [16], [32], while the remaining 20% consisted of N822K mutations. Indeed, *c-KIT* N822K mutations have also been found in testicular GCC [10], [35], [36], indicating a role for this mutation in the development of GCC, independent of the origin in the testis or ovary. Next to these mutations a known synonymous SNP (rs55789615) was detected in exon 17 of case 28, which has been described before in a patient having a N822K mutation in the GCC of the contralateral testis [36]. Besides these, no other aberrations in the exons analyzed could be found in the group of ovarian dysgerminomas. Patients showing expression of c-KIT might benefit from targeted therapy with imatinib mesylate, as has been shown for patients with GIST [37], and also in a patient with metastatic seminoma [38]. This might therefore also be of interest to treat ovarian dysgerminoma.

DSD patients with gonadal dysgenesis or hypovirilization have an increased risk of developing GCC, with GB as the precursor lesion, linked to the presence of (part of) the Y-chromosome. Y-chromosomal material is detected in 90% of patients with dysgenetic gonads, with the *TSPY* gene being seen as the candidate gene in the GonadoBlastoma on the Y-chromosome (GBY) region [39]. Interestingly, the role of TSPY has been suggested to be a repressor of androgen signaling by trapping AR in the cytoplasm, even in presence of the ligand [40]. It is therefore possible that the relative high levels of TSPY protein found in CIS and GB creates a local androgen –insensitivity environment, in which these cells are not able to respond to the presence of ligand. This is of particular interest during the window of so-called mini-puberty, whereby the affected germ cells remain in an embryonic state (positive for OCT3/4 amongst others). During puberty these cells might become sensitive to prolonged and increased levels of androgens and subsequently become invasive (associated by loss of TSPY expression). Here we show that in 89% of cases (17 out of 19 analyzed) where GB was present, either with or without dysgerminoma, positive staining of the TSPY protein could be seen in the neoplastic cells. It is possible that in the two TSPY negative cases (7 and 13) the staining was sub-optimal due to poor tissue fixation, as other markers tested showed unexpected (negative) results (data not shown). In contrast, all cases with ovarian dysgerminoma in a 46,XX (normal female) genetic background were negative for TSPY. The results underline the importance of presence of (part of) the Y-chromosome in the development of GB and point to the fact that in the case of DSD and ovarian dysgerminomas the pathways leading to the tumors are distinct. This is in line with, and extends the results reported by Hoei-Hansen [16], who showed TSPY in five out of seven cases with GB, the precursor lesion of dysgerminoma in DSD patients, and no TSPY protein expression in eleven pure dysgerminoma cases. Presence of the *TSPY* gene in malignant ovarian germ cell tumors has also been studied by Shahsiah *et al*. [41], showing positivity of the gene in 6 out of 47 (12.7%) cases, two patients showed GB and in one patient presence of the Y chromosome was confirmed cytogenetically. However, no *c-KIT* mutation analysis was performed.

Next to the presence of TSPY, also presence of OCT3/4 was investigated in this series. OCT3/4 is one of the key regulators of self renewal and pluripotency of embryonic stem cells, and in normal development this protein is only present in primordial germ cells/gonocytes and oogonia [28], [42]. In the testis expression is only seen in GCC (i.e. seminoma and embryonal carcinoma) and its precursor lesion CIS [25], [43]. In DSD patients OCT3/4 expression is present in GB and dysgerminoma [44], [45]. OCT3/4 was present in all but one GB analyzed in this study, and also a positive staining was found in all dysgerminomas, in line with previous studies [16], [44]. Case 7 which did not show a positive OCT3/4 staining of the GB, also gave mixed results using other markers (negative TSPY staining amongst others), indicating possible poor quality of the material.

Analyzing the presence of *c-KIT* activating - and *PDGFRA* mutations, either by LightCycler melting curve analysis or conventional sequencing, in the group of sixteen DSD cases showing GB, with or without an invasive tumor, showed that in the majority of cases no mutations could be detected (15 out of 16 cases, 94%). It must be mentioned however, that in a number of cases the percentage of tumor present in the sample was low, possibly leading to false negative results. In three patients a shift in melting curve not corresponding to one of the *c-KIT* c.816 mutations investigated was found, and subsequent sequencing of the LightCycler products revealed a wild type exon 17 sequence in case 1 and 2, and a known synonymous SNP (rs 55789615) in case 4 (I798I), although this latter finding was not confirmed by conventional sequencing of the original DNA sample. Strikingly, these three patients all have bilateral disease. The I798I variant was also detected in a patient with ovarian dysgerminoma (case 28, see above). In one patient (case 16) showing GB and mainly dysgerminoma, missense mutations in *c-KIT* were found in exon 9 and 17, resulting in N505I and D820E respectively, which were not present in normal adjacent adnexal material. In this case, which was also positive for TSPY, presence of the Y-chromosome was confirmed with fluorescent *in-situ* hybridization on paraffin embedded material of the dysgerminoma lesion using a Y-centromeric probe (data not shown), confirming a 46,XY-DSD diagnosis. A mutation in *c-KIT* codon 816 in a DSD patient presenting with GB and dysgerminoma has also been reported previously [16], indicating that in rare cases these mutations can be found in DSD patients. Interestingly, the phenotypically male patient described here presented with a unilateral cryptorchid testis, which was removed during orchidopexy. He has two sons, who both presented with bilateral cryptorchid testis, which is one of the major risk factors for testicular GCC [46]. If the mutations found are also present in the sons cannot be ascertained as no material is available for analysis. To our knowledge this is the first time a N505I mutation in exon 9 *of c-KIT* has been found. *c-KIT* mutations in exon 9 have been described in GIST [47], and it is thought that these mutations mimic the conformational change that the extracellular KIT receptor undergoes when SCF is bound [48]. The activating *c-KIT* D820E mutation has been described together with mutations in exon 9, related to sunitinib resistance in GIST [49]. If the mutations found are located on the same or different alleles cannot be determined, as only paraffin embedded material was available for analysis. Besides the specific *c-KIT* c.816 mutations investigated here, other mutations in exon 17 have been reported in GCC; *c-KIT* gain-of-function D820G and Y823D [2], [10], [35], [36] have been found, next to S821F, C809S, Y823N and D816E together with D820H [12], [36] amongst others, which are not present in the cases analyzed here, and thus do not seem to be involved in ovarian dysgerminomas or DSD. Interestingly, recently genome-wide association studies of have identified SNPs within *KITLG* (SCF) as having the strongest association with an increased risk of developing a testicular GCC, pointing to the importance of the SCF-cKIT pathway in this disease [50], [51], [52].

Taken together, *c-KIT* mutations occur in approximately half of pure ovarian dysgerminoma cases, all residing in exon 17, indicating a role in the etiology of the disease. The activated c-KIT, together with prolonged expression of OCT3/4 may allow increased survival and proliferation of undifferentiated gonocytes/oogonia, leading to the development of dysgerminoma. In DSD, presence of Y-chromosomal material leads to the gonadal dysgenesis, in which the germ cells survive because of prolonged expression of both OCT3/4 and TSPY, setting the stage for GB and subsequent dysgerminoma development; although in a minority of cases mutations in *c-KIT* might play a role.

## Supporting Information

Figure S1 **Detection of c-KIT c.816 mutations in patient samples by melting curve analysis.** The y-axis represents fluorescence intensity and the x-axis represents temperature. Mutations lead to different melting temperatures of the hybridization probes from the amplification product. A, B) Melting curves of sample 19 and 22 with and without the addition of LNA are shown together with a positive control harboring the D816V mutation. C) Melting curves of sample 17 with and without the addition of LNA are shown together with a positive control harboring the D816H mutation. D) Melting curves of sample 25 with and without the addition of LNA are shown together with a positive control harboring the D816Y mutation. E, F) Electropherogram showing the A to T mutation in codon 816 in LightCycler products with and without LNA added of sample 19 and 22 respectively. G) Electropherogram showing the G to C mutation in LightCycler products with and without LNA added of codon 816 in sample 17. H) Electropherogram showing the G to T mutation in LightCycler products with and without LNA added of codon 816 in sample 25. Note the suppression of wild type c-KIT and the enrichment of the mutant amplification product in the + LNA samples. pc: positive control, Val: valine mutation, His: histidine mutation, Tyr: tyrosine mutation, LNA: locked nucleic acid.
(TIF)

Table S1 **c-KIT and PDGFRA primers.**
(XLS)
