# Supplementary figures and images for: Prevalence of c-KIT Mutations in Gonadoblastoma and Dysgerminomas of Patients with Disorders of Sex Development (DSD) and Ovarian Dysgerminomas
Source: PLoS One. 2012 Aug 28;7(8):e43952. doi: 10.1371/journal.pone.0043952 (PMC3429439; doi:10.1371/journal.pone.0043952)

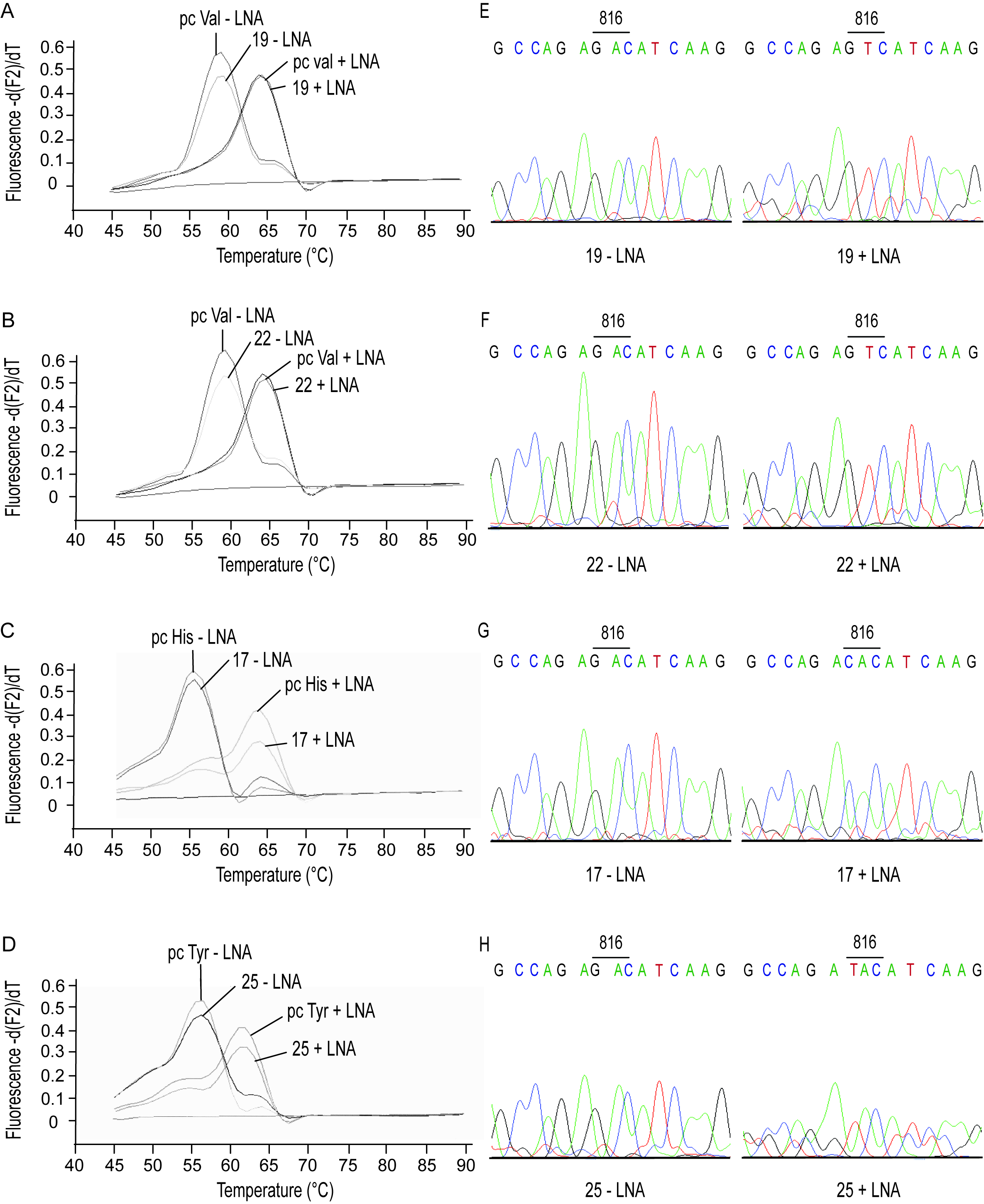

Supplement: Figure S1 — Detection of c-KIT c.816 mutations in patient samples by melting curve analysis. The y-axis represents fluorescence intensity and the x-axis represents temperature. Mutations lead to different melting temperatures of the hybridization probes from the amplification product. A, B) Melting curves of sample 19 and 22 with and without the addition of LNA are shown together with a positive control harboring the D816V mutation. C) Melting curves of sample 17 with and without the addition of LNA are shown together with a positive control harboring the D816H mutation. D) Melting curves of sample 25 with and without the addition of LNA are shown together with a positive control harboring the D816Y mutation. E, F) Electropherogram showing the A to T mutation in codon 816 in LightCycler products with and without LNA added of sample 19 and 22 respectively. G) Electropherogram showing the G to C mutation in LightCycler products with and without LNA added of codon 816 in sample 17. H) Electropherogram showing the G to T mutation in LightCycler products with and without LNA added of codon 816 in sample 25. Note the suppression of wild type c-KIT and the enrichment of the mutant amplification product in the + LNA samples. pc: positive control, Val: valine mutation, His: histidine mutation, Tyr: tyrosine mutation, LNA: locked nucleic acid. (TIF) [file pone.0043952.s001.tif]
